# A Tandem MS Platform for Simultaneous Determination of Urinary Malondialdehyde and Diphenyl Phosphate

**DOI:** 10.3390/ijerph22071130

**Published:** 2025-07-17

**Authors:** Gabriela Chango, Diego García-Gómez, Carmelo García Pinto, Encarnación Rodríguez-Gonzalo, José Luis Pérez Pavón

**Affiliations:** Universidad de Salamanca, Department of Analytical Chemistry, Nutrition and Bromatology, Faculty of Chemical Sciences, 37008 Salamanca, Spain; cristina_chango@usal.es (G.C.); cgp@usal.es (C.G.P.); erg@usal.es (E.R.-G.); jlpp@usal.es (J.L.P.P.)

**Keywords:** hydrophilic interaction liquid chromatography, mass spectrometry, organophosphate flame retardants, restricted access material, oxidative stress

## Abstract

This study presents an advanced analytical method for the simultaneous quantification of malondialdehyde (MDA), a biomarker of oxidative stress, and diphenyl phosphate (DPhP), a metabolite of the organophosphate flame retardant triphenyl phosphate (TPhP), in human urine. The method integrates hydrophilic interaction liquid chromatography (HILIC), a type of liquid chromatography suitable for polar compounds, for MDA separation, and an online restricted access material (RAM), a preconcentration column, for DPhP isolation, achieving high specificity and sensitivity. Validation with certified urine samples confirmed its robustness across diverse analyte concentrations and complex biological matrices. The optimized clean-up steps effectively minimized carryover, allowing for high-throughput analysis. Application to 72 urine samples revealed a significant positive correlation (ρ = 0.702, *p*-value = 1.9 × 10^−7^) between MDA and DPhP levels, supporting a potential link between oxidative stress and TPhP exposure. The subset analysis demonstrated a statistically significant moderate positive correlation in women (ρ = 0.622, *p*-value = 0.020), although this result should be interpreted with caution because of the limited sample size (N = 14). This method provides a powerful tool for biomonitoring oxidative stress and environmental contaminants, offering valuable insights into exposure-related health risks.

## 1. Introduction

Malondialdehyde (MDA) is a well-established biomarker for oxidative stress, indicating lipid peroxidation and cellular damage. Monitoring MDA levels in urine offers a non-invasive way to assess oxidative stress, relevant for evaluating the impact of environmental pollutants and lifestyle factors on health [1,2,3,4]. Elevated MDA concentrations are linked to exposure to toxins and disease development. While enzyme-linked immunosorbent assays are widely used for MDA quantification, their sensitivity and specificity are limited for detecting MDA in complex matrices. Advanced techniques, such as liquid chromatography–tandem mass spectrometry (LC-MS/MS), enhance sensitivity and reliability for detecting low MDA concentrations [5,6,7,8].

Similarly, diphenyl phosphate (DPhP) is a key biomarker for assessing human exposure to triphenyl phosphate (TPhP), a widely used organophosphate flame retardant found in products such as electronics and furniture [9,10]. Measuring DPhP in urine is non-invasive and facilitates large-scale biomonitoring studies, offering a reliable method for assessing internal exposure levels [11]. It helps identify exposure sources and patterns, which is crucial for understanding the health impacts of TPhP, including its potential endocrine-disrupting effects. Due to its low concentration in urine, sensitive methods such as LC-MS/MS are used for accurate detection [12,13,14], with gas chromatography–mass spectrometry (GC-MS) also playing a complementary role in trace analysis [15,16,17,18].

The relationship between oxidative stress and exposure to organophosphate flame retardants (OPFRs), such as TPhP, has been explored in various contexts, highlighting the potential link between biomarkers of exposure and oxidative damage. Although studies directly linking MDA and DPhP are limited, OPFR exposure has been associated with oxidative stress. This connection is of growing concern because of the widespread use of OPFRs in consumer products and their propensity to leach into the environment, leading to significant human exposure.

Chen et al. demonstrated that exposure to TPhP in male mice significantly increased oxidative stress, indicated by elevated MDA levels, alongside endocrine disruption [19]. In pregnant women, Yao et al. reported that exposure to organophosphate ester flame retardants, including TPhP, was associated with thyroid endocrine disruption mediated by oxidative stress pathways. This study found elevated oxidative stress markers, particularly among girls, highlighting sex-specific vulnerabilities to environmental exposures during critical developmental periods [20]. Finally, Guo et al. examined the presence of OPFRs and their metabolites, including DPhP, in paired human blood and urine samples. This study also highlighted the metabolic fate of these compounds, showcasing their persistence and potential for systemic accumulation. By establishing robust links between OPFR exposure and its metabolites in biological matrices, this research laid the groundwork for investigating correlations with oxidative stress markers such as MDA [21].

Given the links between exposure to OPFRs such as TPhP and oxidative stress markers such as MDA, there is a need for advanced analytical methods to support correlation studies. Traditional approaches often involve separate analyses of biomarkers, which are time-consuming and resource-intensive. Rapid techniques such as tandem MS offer exceptional sensitivity, specificity, and throughput, enabling the simultaneous detection of multiple biomarkers in a single run. These innovations streamline analysis, reduce processing times, and improve data reliability, expanding the scope of large-scale biomonitoring studies. MDA and DPhP were selected as representative urinary biomarkers of oxidative stress and organophosphate exposure, respectively, based on their established relevance in environmental health research and their analytical compatibility with LC-MS/MS workflows. Their simultaneous determination allows for a more efficient exploration of potential associations between chemical exposure and oxidative response.

In this context, we developed a rapid and reliable tandem MS method for the simultaneous quantification of MDA and DPhP in urine. We hypothesize that elevated urinary concentrations of DPhP, as a marker of OPFR exposure, are associated with increased levels of MDA, reflecting oxidative stress. The method was applied to explore this relationship and to assess whether such associations differ according to demographic factors such as sex or age.

## 2. Materials and Methods

### 2.1. Chemicals

All chemicals and reagents were of analytical grade and were purchased from commercial suppliers as follows: creatinine (Cre, ≥98%), formic acid (HCOOH), ethylenediamine (EDA, ≥99.5%), ammonium acetate (NH4Ac, ≥97%) and malondialdehyde tetrabutylammonium salt (≥96%) were purchased from Sigma Aldrich (Steinheim, Germany). DPhP (98%) was supplied by Cymit (Pamplona, Spain). Acetonitrile (ACN, 99.9%) was purchased from Fisher Scientific (Pittsburgh, PA, USA), sodium hydroxide (NaOH, 98%) and hydrochloric acid (HCl, 99%) from Panreac (Barcelona, Spain). Picric acid was purchased from D’hemio (Madrid, Spain). Certified urine samples were provided from Centre de Toxicologie du Québec (Quebec, Canada). Ultra-high quality (UHQ) water used was obtained with a Wasserlab Ultramatic purification system (Noain, Spain).

### 2.2. Standard Solutions

Stock solutions of MDA (100 mg·mL^−1^) and DPhP (500 mg·mL^−1^) were prepared in UHQ water and stored at 4 °C in brown glass bottles. Working solutions were freshly prepared each day by appropriate dilution of the stock solutions in UHQ water.

### 2.3. Sample Collection and Treatment

Urine samples collected from sixty-one volunteers (numbered from U1 to U61) were used for the development and validation of the method. Table 1 summarizes demographic information for the volunteers, including gender, age, and smoking habits, which were recorded through an oral questionnaire during recruitment. All participants provided written informed consent prior to sample collection. Additionally, 11 certified urine samples provided by the Centre de Toxicologie du Québec (designated from U2204 to U2201) were included for method validation and overall correlation analysis. These samples correspond to proficiency testing materials containing unspiked known concentrations of the target analytes, selected to reflect typical urinary levels observed in real exposure scenarios. However, they were excluded from stratified correlation analyses by sex and age due to the absence of demographic information, which would hinder accurate subgroup classification and could compromise the validity of such comparisons.

After collection, the samples were immediately frozen and stored until analysis and thawed at room temperature before being analyzed. The sample treatment procedures for MDA and DPhP were based on previously established protocols developed by our group [7,22], which ensured analytical consistency and continuity with earlier validated methods.

Quantification was carried out by means of a one-point standard addition method.

To compensate for variations in urine concentrations, MDA and DPhP concentrations were normalized against creatinine (mg·g^−1^ Cre and ng·g^−1^ Cre, respectively). Urinary creatinine levels were determined with the Jaffe method [23], based on the reaction between creatinine and picric acid, using photometric detection.

### 2.4. Instrumentation

The LC–MS/MS system consisted of an Agilent 1200 series HPLC (Agilent Technologies, Waldbronn, Germany) equipped with a binary pump, an isocratic pump, a membrane degasser, an autosampler, a 500 µL injection loop, a six-port valve, and a 6410 LC/MS QqQ mass spectrometer with an electrospray ionization (ESI) source. The nebulizer pressure was set at 35 psi, the voltage at ±3500 V, and nitrogen was used as the drying (12 L·min^−1^, 350 °C) and collision gas.

For MDA analysis, separation was achieved using an Accucore Urea-HILIC column (150 × 2.1 mm, 2.6 µm particles, Thermo Scientific, Waltham, MA, USA). The quantification transition was 71 → 41 (collision energy (CE): 10 eV), with ESI in negative mode, a fragmentor voltage of 90 V, and a dwell time of 200 ms.

For DPhP, an online RAM-based isolation and concentration approach was used with a Shim-pack MAYI-ODS column (10 × 4.6 mm, 50 µm, SHIMADZU, Kyoto, Japan). Detection was conducted using the same mass spectrometry system in negative ESI mode, with a fragmentor voltage of 130 V, dwell time of 200 ms, and a quantification transition of 249 → 93 (CE: 28 eV).

### 2.5. Instrumental Setup

**Figure 1:**
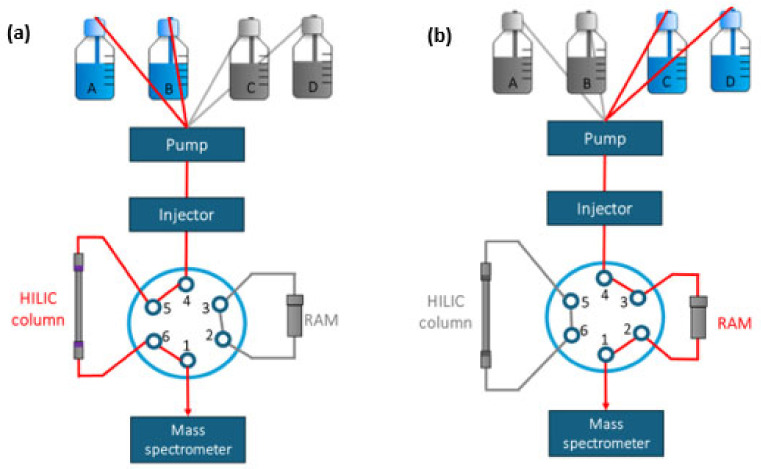
Instrumental setup of the LC-MS/MS method. (**a**) Elution position for MDA analysis: Accucore Urea-HILIC column; mobile phase: ACN, HCOOH 25 mM aqueous solution (93:7 (*v*/*v*)); (**b**) elution position for DPhP analysis: RAM (shim-pack MAYI-ODS); loading solvent: 20 mM NH_4_Ac in UHQ water, elution solvent: ACN:UHQ (99:1 (*v*/*v*)). Abbreviations: LC-MS/MS: liquid chromatography–tandem mass spectrometry; MDA: malondialdehyde; HILIC: hydrophilic interaction liquid chromatography; ACN: acetonitrile; HCOOH: formic acid; DPhP: diphenyl phosphate; RAM: restricted access material; NH_4_Ac: ammonium acetate; UHQ: ultra-high quality.

Figure 1 illustrates the instrumental setup. With the six-port valve in position 1–6 (Figure 1a), 5 µL of treated urine was injected, and the pump was immediately started to deliver the solvent for MDA determination (A: ACN aqueous solution, B: 25 mM HCOOH (93:7 (*v*/*v*), pH = 3.72)) at a flow rate of 0.4 mL·min^−1^ under isocratic conditions for 7 min. During this step, matrix components from the urine were removed while MDA was retained on the HILIC column. An additional 7 min clean-up step with 25 mM ACN:HCOOH aqueous solution (50:50 (*v*/*v*)) was conducted.

At minute 15, 500 µL of the sample was injected, the valve position was switched to position 1-2 (Figure 1b), and the solvent gradient (C: 20 mM NH4Ac in UHQ water, D: ACN:UHQ (99:1 (*v*/*v*))) was initiated. The gradient was programmed as follows: start at 100% C, hold for 1 min, transition to 100% D over 1.02 min, and hold for 9 min. DPhP was eluted from the RAM, where it was isolated and preconcentrated, at a flow rate of 0.5 mL·min^−1^.

At minute 30, the procedure was terminated, and the gradient and six-port valve were returned to their initial conditions. A post-run program was initiated, maintaining the system at the initial conditions to equilibrate for the next analysis. This joint method is designed as a fully automated sequence, allowing for the continuous and uninterrupted analysis of multiple samples without the need for manual intervention or instrumental reconfiguration. By integrating both MDA and DPhP analysis into a single, streamlined workflow, the method not only enhances reproducibility and reduces potential human error but also significantly improves throughput, making it highly suitable for large-scale biomonitoring studies.

### 2.6. Statistical Analysis and Correlation Studies

Initially, a power analysis was run to calculate the appropriate number of samples. Assuming a pre-hoc medium correlation (ρ = 0.5), to obtain a type I error of 5% and a power of 90%, at least 37 samples had to be analyzed. Keeping in mind a drop-out of ca. 50% for DPhP, based on its prevalence determined by previous studies [10,21,22], around 70 samples must be processed. Only samples with DPhP concentrations above LOQ were considered for correlation studies. Samples below the LOD were excluded and not subjected to imputation or substitution.

All urinary concentrations of MDA and DPhP were creatinine-adjusted before statistical analysis. These concentrations exhibited a non-normal distribution according to the Shapiro–Wilk test, with *p*-values of 1.0 × 10^−11^ and 5.7 × 10^−11^, respectively. After log transformation, the *p*-values increased to 0.14 and 0.28, indicating a log-normal distribution. Despite this, Spearman’s rank correlation was chosen over Pearson’s correlation because of its robustness against outliers, its suitability for small sample sizes, and the fact that the relationship between MDA and DPhP was not assumed to be strictly linear. Spearman’s correlation coefficient (ρ) was therefore calculated using log-transformed data. A *p*-value < 0.05 was regarded as statistically significant. Data analyses were all performed using RStudio (Version: 2024.12.0+467).

## 3. Results and Discussion

### 3.1. Analytical Performance

The combined tandem MS method demonstrated remarkable performance for the simultaneous detection of MDA and DPhP in urine samples. The use of separate mechanisms for MDA and DPhP ensured no overlap or interference between the analytes, enhancing precision and reliability. Quantification for both analytes was conducted using a one-point standard addition approach, which is well-suited for endogenous compounds [24]. Continuous monitoring of instrumental performance was achieved by injecting MDA (25 ng·mL^−1^) and DPhP (10 ng·mL^−1^) standards every 15 injections. To further validate the accuracy of the method, certified samples for DPhP, provided by the Centre de Toxicologie du Québec, were analyzed as part of this study. These reference samples served as an additional quality control measure, reinforcing the reliability of the developed methodology. The limit of detection (LOD) and limit of quantification (LOQ) values used in this study were taken from previously validated methods developed by our group. For MDA, the LOD and LOQ were 0.20 ng·mL^−1^ and 0.67 ng·mL^−1^, respectively, as established in Chango et al. [7]. For DPhP, the LOD and LOQ were 0.03 ng·mL^−1^ and 0.1 ng·mL^−1^, respectively, based on the method described in Chango et al. [22].

**Figure 2:**
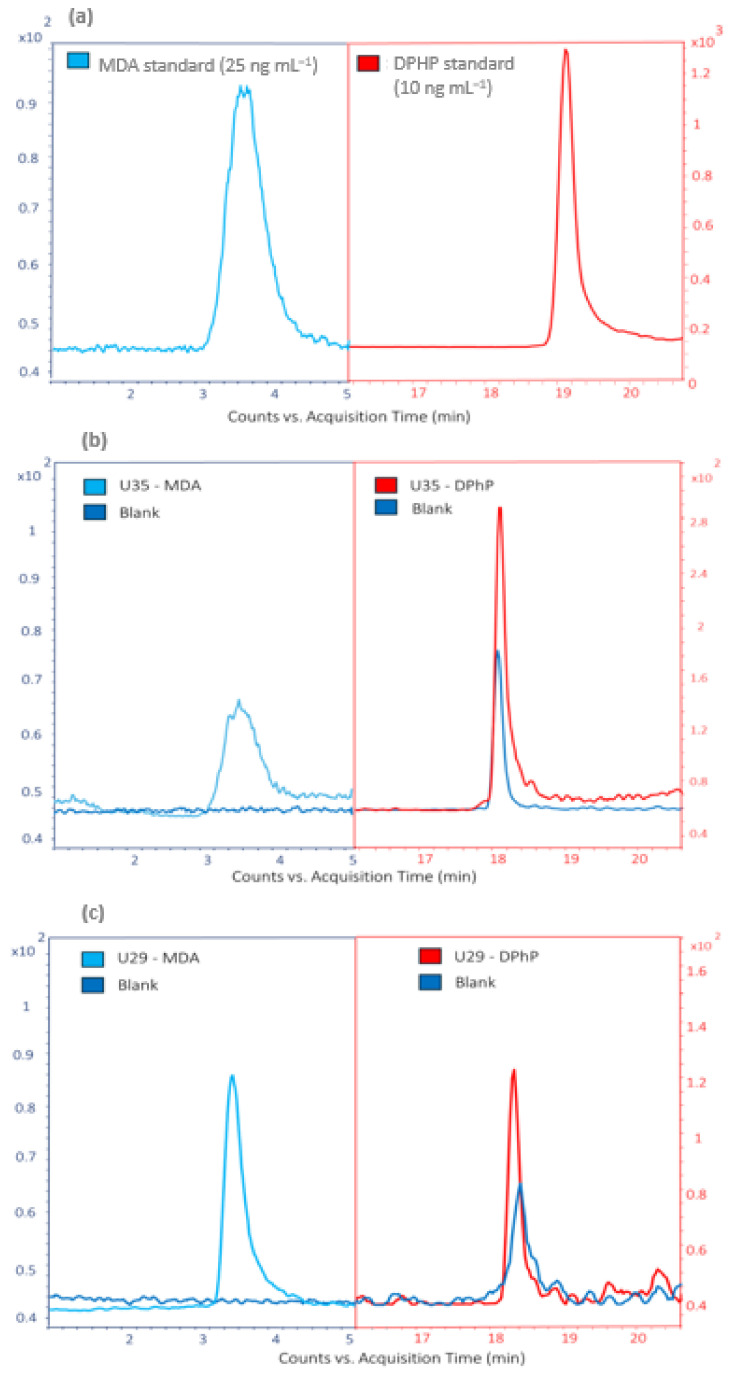
Chromatograms obtained from standards and urine samples for MDA and DPhP using the LC-MS/MS system. (**a**) Comparison of a 25 ng·mL^−1^ MDA standard and 10 ng·mL^−1^ of DPhP standard; (**b**) signals for MDA and DPhP for sample U35; (**c**) signals for MDA and DPhP for sample U29. Colors: MDA (light blue), DPhP (red), blank (blue). Abbreviations: MDA: malondialdehyde; DPhP: diphenyl phosphate; LC-MS/MS: liquid chromatography–tandem mass spectrometry.

Representative chromatograms further illustrate the performance of the method. Figure 2a shows injections of MDA and DPhP standards used for monitoring. Figure 2b displays the chromatogram for sample U35, which exhibited the highest DPhP concentration but a low MDA level. Figure 2c presents the chromatogram for sample U29, the third highest in MDA concentration but with a low DPhP level. Notably, a detectable DPhP signal was observed in the blanks, suggesting potential environmental contamination or degradation of TPhP. To address this, process blanks were regularly analyzed, and DPhP concentrations in urine samples were corrected by subtracting the average blank signal, ensuring accurate quantification. For MDA, the blank signal was negligible, ensuring accurate quantification without significant background interference.

One potential drawback of online extraction columns for high-throughput analyses is the risk of carryover between samples. To assess this, a blank sample was injected after the analysis of 10 consecutive urine samples. The results confirmed no significant carryover for either analyte, demonstrating the effectiveness of the clean-up and equilibration steps incorporated into the method. This finding further reinforces the robustness and reliability of the analytical approach.

### 3.2. MDA and DPhP Concentrations

The method was successfully applied to 72 urine samples, which were analyzed in triplicate to ensure reliability. To account for urinary dilution [25], concentrations were normalized against creatinine levels, as summarized in Table 1. The results revealed variability in analyte concentrations across individuals, with samples U31 and U35 exhibiting notably high levels of MDA and DPhP, respectively, highlighting the capacity of the method to handle diverse concentration ranges.

The geometric mean (GM) urinary MDA concentration in this study was 1.2 mg·g^−1^ Cre. While some studies, such as Toto et al. [1], report lower GMs, these differences likely reflect variations in population characteristics, exposure to oxidative stressors, and methodological approaches.

Regarding DPhP, our findings (median: 252 ng·g^−1^ Cre, N = 39) are consistent with levels reported by Li et al. [26] (median: 230 ng·g^−1^ Cre, N = 46), suggesting similar exposure patterns across populations. DPhP was detected in 54.2% of samples, comparable to the 62% prevalence reported by Dodson et al. [27]. These results highlight the widespread exposure to DPhP and the variability in concentrations influenced by environmental, lifestyle, and methodological factors.

The results presented in Table 2 highlight differences in MDA and DPhP concentrations across age and sex subgroups. Regarding age, the sample was stratified using a cutoff age of 37 years (median). MDA concentrations were relatively low across all groups, with GM ranging from 0.95 mg·g^−1^ Cre (95% CI: 0.46–1.95) in the younger age group (≤37 years) to 1.41 mg·g^−1^ Cre (95% CI: 0.89–2.22) in the older group (>37 years). Similarly, sex-based analysis indicates comparable MDA levels between women (GM: 1.15 mg·g^−1^ Cre, 95% CI: 0.62–2.12) and men (GM: 1.25 mg·g^−1^ Cre, 95% CI: 0.72–2.18), suggesting minimal sex-specific differences in oxidative stress biomarkers.

In contrast, DPhP concentrations exhibited greater variability, with higher levels observed in the older age group (GM: 19.14 ng·g^−1^ Cre, 95% CI: 7.44–49.23) compared with the younger group (GM: 12.65 ng·g^−1^ Cre, 95% CI: 6.85–23.34). The wide confidence intervals for DPhP reflect substantial interindividual variability, likely because of differences in environmental exposure or metabolic rates [28]. Sex-based differences were less pronounced, with women showing slightly higher DPhP levels (GM: 17.23 ng·g^−1^ Cre, 95% CI: 7.32–40.58) than men (GM: 14.04 ng·g^−1^ Cre, 95% CI: 6.33–31.18). The absence of detectable DPhP in some samples is attributed to concentrations below the LOD of our method, reflecting intermittent exposure to organophosphate flame retardants.

### 3.3. Correlation Study

As stated in the materials and methods section, normality was checked by Shapiro–Wilk (S-W) tests showing *p*-values for creatinine-adjusted concentrations of MDA and DPhP of 10^−11^ and 5.7 × 10^−11^, respectively. Nonetheless, log-transformed data indicate *p*-values of 0.14 and 0.28, which indicate a log-normal distribution for both urinary concentrations. Subsequently, Spearman’s correlation analyses were run for log-transformed data to assess the relationship between the urinary levels of the oxidative biomarker MDA and DPhP, a metabolite linked to the exposure to the ubiquitous organophosphate flame retardant TPhP (Table 1). A ρ of 0.70243 was found (ρ = 0.4898–0.8362 at a 95% level of significance) with a corresponding *p*-value of 1.9 × 10^−7^. This suggests a significant correlation between the urinary concentration of MDA and DPhP, which corroborates previous findings in both animal and human studies. For instance, experimental studies in mice have demonstrated that TPhP induces oxidative stress and endocrine disruption [19,29]. Similarly, in humans, epidemiological studies have reported positive associations between OPFR metabolites and oxidative stress biomarkers [30,31,32]. Finally, Guo et al. documented that exposure to a mixture of OPFRs is associated with increased MDA levels in paired human blood and urine samples [21]. However, it is important to note that the relationship between OPFR exposure and oxidative stress may be influenced by factors such as metabolic variability, coexposure to other environmental contaminants, and individual susceptibility.

**Figure 3:**
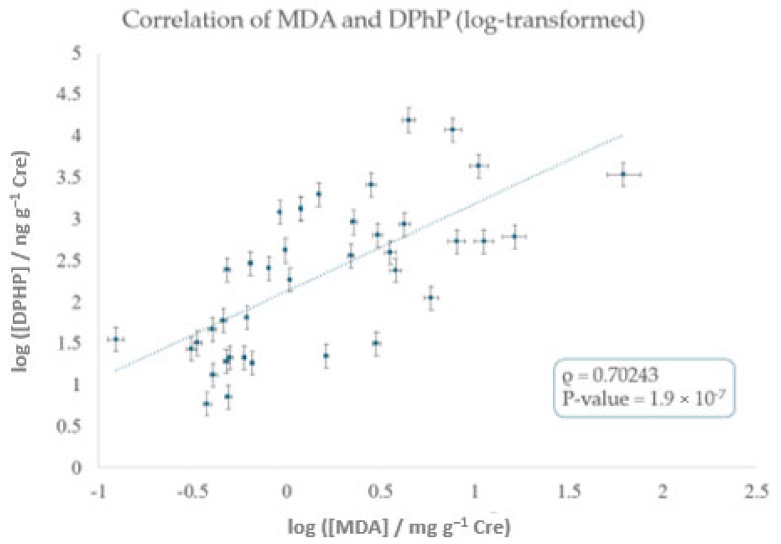
Scatter plot of creatinine-adjusted urinary concentrations of MDA and DPhP, both log-transformed. The dotted line represents a linear regression fit applied to all samples with detectable levels of DPhP (N = 39). A positive association was found (Spearman’s ρ = 0.702; *p*-value = 1.9 × 10^−7^). Abbreviations: MDA: malondialdehyde; DPhP: diphenyl phosphate; Cre: creatinine.

Figure 3 plots log-transformed concentrations of MDA vs. those of DPhP for the positive 39 urine samples. It should be highlighted that, according to power analysis, 39 samples assured a power of 91.5%, with a type I error of 0.05.

**Figure 4:**
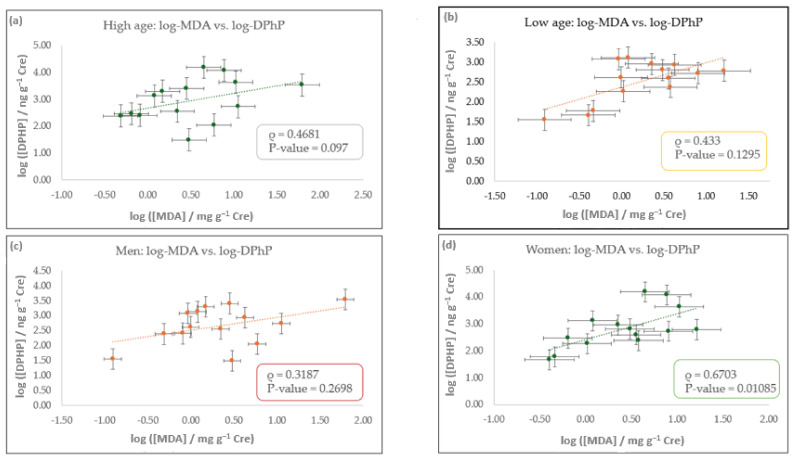
Scatter plots of log-transformed urinary concentrations of MDA and DPhP, adjusted by creatinine, stratified by age and sex. Subplots show the correlations for: (**a**) participants aged ≥37 years (high-age group), (**b**) participants <37 years (low-age group), (**c**) male participants, and (**d**) female participants. Each panel includes a linear regression fit (dotted line) with 95% confidence intervals represented as error bars. Spearman’s correlation coefficients (ρ) and corresponding *p*-values are shown within each subplot. Abbreviations: MDA: malondialdehyde; DPhP: diphenyl phosphate; Cre: creatinine.

To further investigate this association, Spearman’s correlation analyses were conducted on log-transformed data within subsets based on age (low and high) or sex (male and female). Only samples U1–U61 with detectable DPhP were included, excluding certified reference samples. This stratified approach aimed to assess potential differences in correlation patterns across demographic groups. The demographic characteristics of this study’s population, including sex, age, and smoking habits, are summarized in Table 1 and were used to define the stratification criteria. Figure 4 presents scatter plots of log-transformed urinary MDA and DPhP concentrations, stratified by sex or age group using a median cutoff of 37 years.

In the high-age group (≥37 years, N = 14), MDA and DPhP indicate a positive correlation (ρ = 0.4681; *p*-value = 0.097; ρ = −0.1823-0.8336 at a 95% level of significance), although it did not reach statistical significance. In the low-age group (<37 years, N = 14), a similar trend was observed (ρ = 0.433; *p*-value = 0.1295; ρ = −0.2198–0.8179 at a 95% level of significance). In both groups, the lack of significance may be due to the small sample size, which reduces statistical power to 65%. Therefore, further studies should be carried out.

Concerning sex-based stratification, the sample was divided into men and women. Among men (N = 14), the correlation was again positive but not conclusive (ρ = 0.3187; *p*-value = 0.2698; ρ = −0.3304–0.7631 at a 95% level of significance). In contrast, in the women’s group (N = 14), a moderate association was observed between MDA and DPhP (ρ = 0.6703; *p*-value = 0.01085; ρ = 0.08414–0.9118 at a 95% level of significance), indicating a statistically supported relationship. This association may reflect sex-specific differences in exposure patterns and metabolic pathways [21,33,34]. However, further studies with larger sample sizes are needed to confirm these findings.

Although additional demographic data, such as smoking status, were collected, only age and sex were included in the present exploratory analysis. Further studies could integrate additional covariates to improve the interpretation of exposure patterns and potential confounding effects.

## 4. Conclusions

This study introduces a tandem MS methodology for the simultaneous analysis of MDA and DPhP in urine samples. The method combines distinct separation mechanisms—HILIC for MDA and online RAM extraction for DPhP—ensuring accurate quantification without mutual interference. Its performance in complex matrices, effective control of carryover, and high-throughput capacity demonstrate its suitability for biomonitoring applications.

The results revealed a positive association between urinary levels of DPhP and MDA, supporting a possible link between exposure to organophosphate flame retardants and oxidative stress. This association was statistically supported in women, suggesting potential sex-specific differences in exposure or metabolism. In contrast, no consistent correlations were observed in men or across age groups, likely due to the limited sample size.

Further studies with larger and more diverse cohorts are needed to confirm these findings and better understand the mechanisms linking oxidative stress and organophosphate exposure.

## Figures and Tables

**Table 1:** Urinary concentrations of MDA and DPhP in samples (U1–U61) and certified urine samples (U2204–U2201).

Sample	Sex	Age	[Cre] mg·dL^−1^	[MDA] ng·mL^−1^	[MDA] mg·g^−1^ Cre	[DPhP] ng·mL^−1^	[DPhP] ng·g^−1^ Cre
U1	Woman	19	145	3600 ± 300	2.5 ± 0.2	0.9 ± 0.7	600 ± 400
U2	Man	55	53	5900 ± 200	11.0 ± 0.4	0.3 ± 0.2	500 ± 300
U3	Woman	36	48	1500 ± 200	3.0 ± 0.3	0.3 ± 0.1	700 ± 200
U4	Man	37	20	340 ± 90	1.7 ± 0.5	<LOD	<LOD
U5	Woman	78	11	550 ± 80	5.1 ± 0.8	<LOD	<LOD
U6	Man	87	49	700 ± 100	1.3 ± 0.3	<LOD	<LOD
U7	Man	52	285	8600 ± 600	3.0 ± 0.2	0.09 ± 0.03	31 ± 9
U8	Woman	22	13	360 ± 80	2.9 ± 0.6	<LOD	<LOD
U9	Man	86	80	650 ± 50	0.82 ± 0.06	<LOD	<LOD
U10	Woman	81	15	630 ± 30	4.1 ± 0.2	<LOD	<LOD
U11	Man	54	104	1540 ± 90	1.48 ± 0.09	2.1 ± 0.3	2000 ± 300
U12	Woman	48	29	150 ± 40	0.5 ± 0.1	<LOD	<LOD
U13	Woman	32	101	3800 ± 200	3.8 ± 0.2	0.24 ± 0.04	240 ± 40
U14	Woman	35	30	4900 ± 300	16 ± 1	0.18 ± 0.03	600 ± 100
U15	Woman	52	77	1900 ± 100	2.4 ± 0.2	<LOD	<LOD
U16	Man	39	149	8800 ± 300	5.9 ± 0.2	0.17 ± 0.03	110 ± 20
U17	Man	53	69	1600 ± 100	2.3 ± 0.2	<LOD	<LOD
U18	Woman	39	15	1720 ± 70	11.3 ± 0.5	<LOD	<LOD
U19	Man	47	42	210 ± 70	0.5 ± 0.1	<LOD	<LOD
U20	Man	58	90	1200 ± 300	1.3 ± 0.3	<LOD	<LOD
U21	Woman	56	23	60 ± 40	0.3 ± 0.2	<LOD	<LOD
U22	Woman	28	103	50 ± 30	0.05 ± 0.03	<LOD	<LOD
U23	Man	32	263	110 ± 50	0.04 ± 0.02	<LOD	<LOD
U24	Woman	24	115	60 ± 40	0.06 ± 0.04	<LOD	<LOD
U25	Man	38	123	220 ± 80	0.18 ± 0.06	<LOD	<LOD
U26	Man	51	99	1100 ± 200	1.1 ± 0.2	<LOD	<LOD
U27	Woman	31	14	7000 ± 1000	49 ± 9	<LOD	<LOD
U28	Woman	39	198	1600 ± 100	0.79 ± 0.05	<LOD	<LOD
U29	Man	66	52	14,000 ± 2000	27 ± 3	<LOD	<LOD
U30	Man	39	64	290 ± 60	0.45 ± 0.09	<LOD	<LOD
U31	Man	64	33	20,000 ± 2000	62 ± 5	1.13 ± 0.06	3400 ± 200
U32	Woman	43	14	1500 ± 100	10.5 ± 0.9	0.6 ± 0.1	4300 ± 700
U33	Woman	50	57	360 ± 60	0.60 ± 0.10	0.17 ± 0.07	300 ± 100
U34	Man	45	12	340 ± 50	2.8 ± 0.4	0.31 ± 0.01	2500 ± 100
U35	Woman	64	15	670 ± 50	4.5 ± 0.3	2.3 ± 0.3	15,000 ± 2000
U36	Woman	9	13	290 ± 40	2.3 ± 0.3	0.12 ± 0.05	900 ± 400
U37	Woman	41	8	650 ± 20	7.7 ± 0.3	1.0 ± 0.2	12,000 ± 200
U38	Woman	35	10	340 ± 40	3.6 ± 0.4	0.04 ± 0.02	400 ± 200
U39	Woman	72	4	136 ± 6	3.4 ± 0.1	<LOD	<LOD
U40	Man	37	53	310 ± 50	0.59 ± 0.09	<LOD	<LOD
U41	Man	44	13	3100 ± 80	23.4 ± 0.6	<LOD	<LOD
U42	Woman	43	6	200 ± 20	3.5 ± 0.3	<LOD	<LOD
U43	Man	3	35	1480 ± 70	4.2 ± 0.2	0.30 ± 0.05	900 ± 100
U44	Man	38	15	340 ± 30	2.2 ± 0.2	0.05 ± 0.02	400 ± 100
U45	Man	38	21	250 ± 90	1.2 ± 0.7	0.28 ± 0.08	1300 ± 400
U46	Man	51	18	340 ± 40	1.9 ± 0.2	<LOD	<LOD
U47	Man	15	26	240 ± 50	0.9 ± 0.2	0.32 ± 0.04	1200 ± 200
U48	Woman	14	26	310 ± 40	1.2 ± 0.2	0.34 ± 0.04	1300 ± 200
U49	Man	17	38	380 ± 30	0.99 ± 0.06	0.16 ± 0.05	400 ± 100
U50	Man	49	26	1300 ± 100	5.1 ± 0.4	0.42 ± 0.08	250 ± 50
U51	Woman	53	38	70 ± 30	0.17 ± 0.09	<LOD	<LOD
U52	Man	39	84	360 ± 50	0.43 ± 0.06	<LOD	<LOD
U53	Woman	24	166	290 ± 40	0.17 ± 0.02	<LOD	<LOD
U54	Woman	34	86	700 ± 50	0.81 ± 0.06	<LOD	<LOD
U55	Man	55	84	1060 ± 40	1.26 ± 0.05	0.5 ± 0.1	240 ± 50
U56	Woman	25	76	1520 ± 90	2.0 ± 0.1	0.20 ± 0.05	60 ± 10
U57	Man	31	101	290 ± 10	0.29 ± 0.01	0.08 ± 0.03	25 ± 9
U58	Woman	29	218	450 ± 60	0.21 ± 0.03	<LOD	<LOD
U59	Woman	29	330	110 ± 40	0.03 ± 0.01	<LOD	<LOD
U60	Woman	28	235	570 ± 70	0.24 ± 0.03	0.06 ± 0.01	130 ± 10
U61	Woman	27	111	1310 ± 30	1.18 ± 0.03	0.23 ± 0.03	160 ± 20
U2204	NA	NA	146	870 ± 90	0.6 ± 0.1	0.03 ± 0.02	21 ± 9
U2206	NA	NA	119	490 ± 50	0.41 ± 0.06	0.01 ± 0.01	13 ± 9
U2106	NA	NA	93	310 ± 40	0.34 ± 0.06	0.03 ± 0.01	30 ± 10
U2205	NA	NA	143	450 ± 60	0.31 ± 0.01	0.04 ± 0.02	30 ± 10
U2303	NA	NA	137	650 ± 50	0.48 ± 0.05	0.03 ± 0.02	19 ± 8
U2203	NA	NA	131	490 ± 30	0.38 ± 0.01	0.01 ± 0.01	6 ± 5
U2104	NA	NA	119	730 ± 50	0.61 ± 0.09	0.08 ± 0.02	60 ± 20
U2302	NA	NA	118	580 ± 30	0.49 ± 0.09	0.01 ± 0.01	7 ± 6
U2301	NA	NA	113	1830 ± 30	1.62 ± 0.07	0.02 ± 0.01	20 ± 10
U2202	NA	NA	93	460 ± 80	0.50 ± 0.06	0.02 ± 0.01	20 ± 10
U2201	NA	NA	79	520 ± 30	0.66 ± 0.06	0.01 ± 0.01	18 ± 9

Abbreviations: MDA: malondialdehyde; DPhP: diphenyl phosphate; Cre: creatinine; LOD: limit of detection; NA: not available; demographic data not provided for certified samples.

**Table 2:** Geometric mean (GM) and 95% confidence intervals (CI) of urinary MDA and DPhP concentrations stratified by age and sex.

Category		MDA			DPhP		
		N ^a^	GM	95% CI	N ^a^	GM	95% CI
Age ^b^	High	36	1.41	0.89–2.22	14	19.14	7.44–49.23
	Low	25	0.95	0.46–1.95	14	12.65	6.85–23.34
Sex	Woman	32	1.15	0.62–2.12	14	17.23	7.32–40.58
	Man	29	1.25	0.72–2.18	14	14.04	6.33–31.18

^a^ Number of samples included in the analysis (Note: N (MDA) ≠ N (DPhP) due to only including samples with detectable DPhP values); ^b^ Median age cutoff: 37 years. Abbreviations: MDA: malondialdehyde; DPhP: diphenyl phosphate.

## Data Availability

The original contributions presented in this study are included in the article; further inquiries can be directed to the corresponding author.
